# Diagnostic Methods for Clinical Lumbar Instability: A Systematic Review of Current Status, Challenges, and Future Directions

**DOI:** 10.7759/cureus.108817

**Published:** 2026-05-13

**Authors:** Mebin S Thomas, Sandeep Shinde, Neha S Chaudhary, Sachin Chaudhary

**Affiliations:** 1 Musculoskeletal Sciences, Krishna College of Physiotherapy, Krishna Vishwa Vidyapeeth, Deemed to be University, Karad, IND; 2 Neuroscience, Datta Meghe College of Physiotherapy, Nagpur, IND; 3 Cardiovascular and Respiratory Physiotherapy, Datta Meghe College of Physiotherapy, Nagpur, IND

**Keywords:** diagnostic techniques and procedures, low back pain, lumbar vertebrae, radiography, spinal instability

## Abstract

Lumbar segmental instability is characterized by abnormal vertebral movement, resulting in localized pain and restricted function. Despite its clinical significance, the lack of diagnostic standards leads to widespread practice variability. This review critically analyzes existing diagnostic protocols, identifies methodological shortcomings, and synthesizes multimodal diagnostic strategies.

Adhering to Preferred Reporting Items for Systematic Reviews and Meta-Analyses (PRISMA) 2020 guidelines, systematic searches across PubMed, Scopus, Web of Science, Cochrane, and Embase (inception to December 2025) targeted human diagnostic and observational studies, explicitly excluding studies that used artificial intelligence (AI) algorithms. Of the 657 records, 12 met the eligibility criteria. Quality was appraised using the Quality Assessment of Diagnostic Accuracy Studies-2 (QUADAS-2), Newcastle-Ottawa Scale (NOS), and Prediction Model Risk of Bias Assessment Tool (PROBAST). Given the clinical heterogeneity, a thematic narrative synthesis was conducted following the Synthesis Without Meta-analysis (SWiM) guidelines.

Findings confirm no single test is adequate. The Passive Lumbar Extension and Lumbar Rocking tests demonstrated strong screening potential (sensitivities: 84.2% and 95.56%; specificities: 90.4% and 40%). Clustered clinical testing significantly improved precision (LR+ 5.80). For imaging, dynamic assessments, such as 3-kg weight-lifting flexion radiographs (88% detection at L3/L4) and sit-to-stand kinematics, outperformed static images. Advanced predictive models using 3D-CT and least absolute shrinkage and selection operator (LASSO) regression achieved exceptional theoretical accuracy (area under the curve (AUC) 0.972); however, alongside tools such as the Jakarta Instability Score, they carry a high risk of statistical bias and currently lack independent external validation.

Diagnosing lumbar instability requires a comprehensive multimodal strategy. Current evidence precision is heavily constrained by small cohort sizes and a pervasive lack of 95% confidence intervals. Future research must prioritize externally validated predictive models and standardized imaging reference criteria.

## Introduction and background

Abnormal vertebral movement patterns define lumbar segmental instability, a multifaceted condition that can lead to neurological issues, restricted function, and localized pain [1]. Within the population of patients with chronic, non-specific low back pain, spinal instability is a frequent underlying factor, with estimated prevalence rates ranging between 12% and 40% [2]. This wide variance in epidemiological reporting is largely due to the absence of a universally accepted diagnostic gold standard and to differing criteria used across studies, ranging from purely structural radiographic thresholds to entirely symptom-based clinical diagnoses.

Lumbar segmental instability typically develops in the context of degenerative changes affecting the intervertebral disc, facet joints, and supporting ligamentous structures. Progressive disc degeneration leads to loss of disc height and altered load distribution, increasing stress on posterior elements and potentially resulting in facet joint arthropathy (degeneration of the posterior spinal joints) and ligamentous laxity (abnormal loosening of the supporting ligaments) [3]. This breakdown of the spinal joints and discs leads to varying degrees of instability, ranging from minor muscle control issues to severe conditions such as spondylolisthesis, in which one vertebra slips forward over another.

From a clinical standpoint, individuals with lumbar segmental instability frequently report mechanical low back discomfort that is aggravated during extension-based movements, sustained walking, or prolonged standing. Patients often describe characteristic symptoms of instability, such as a subjective feeling of the lumbar spine "giving way" during movement. These clinical presentations frequently necessitate the use of compensatory strategies. Some patients also report a "catching" sensation during trunk movement or apprehension regarding specific movements due to anticipated pain or instability [4].

According to the foundational definition proposed by White and Panjabi, clinical instability occurs when the spine loses its ability to maintain proper alignment and movement patterns under everyday physiological loads, thereby posing a risk of neurological compromise, progressive deformity, or incapacitating pain [5]. This definition highlights the functional consequences of abnormal motion rather than focusing solely on quantitative displacement measurements.

The theoretical understanding of this condition has evolved considerably since these early biomechanical descriptions. Panjabi's highly influential neutral-zone model conceptualizes instability as increased laxity within the "neutral zone," the initial range of intervertebral motion where the spine encounters minimal resistance (often visualized conceptually as a marble resting in a shallow bowl). This abnormal laxity arises from a failure or dysfunction across three interdependent stabilizing subsystems: the active system (muscular support), the passive system (ligamentous and osseous structures), and the neural system (proprioceptive feedback and motor control) [6]. This model highlights that instability is not simply excessive movement but a fundamental failure of the spine to preserve controlled motion.

Contemporary literature distinguishes between mechanical (radiographic) instability and functional (clinical) instability. It is important to acknowledge that while these conditions often coexist, they constitute separate clinical entities with distinct diagnostic markers [7]. Mechanical instability refers to objectively measurable excessive motion on imaging studies, typically quantified through translational and angular displacement on dynamic radiographs. Functional instability, conversely, describes symptomatic instability characterized by aberrant movement patterns, motor control deficits, and pain provocation during specific activities, which may occur with or without radiographic evidence of excessive motion [6,7].

The clinical significance of accurately diagnosing lumbar instability extends beyond academic interest to fundamental treatment decisions. Identification of instability influences choices between conservative management strategies, including targeted stabilization exercises, manual therapy, bracing, and surgical interventions such as spinal fusion [8].

However, the lack of universally accepted diagnostic standards has led to considerable variation in clinical practice and difficulty in comparing research outcomes across studies. To address this diagnostic gap, this systematic review was conducted with the following specific objectives: (1) to critically analyze the efficacy of existing diagnostic protocols for lumbar instability, (2) to identify inherent methodological and clinical shortcomings of these isolated tests, and (3) to investigate and synthesize more robust, multimodal strategies for future clinical evaluation.

## Review

Methods

Adhering to the Preferred Reporting Items for Systematic Reviews and Meta-Analyses (PRISMA) 2020 standards [9], a prospective protocol could not be registered with PROSPERO, as the registry's specific module for “diagnostic test accuracy” reviews is currently under development and unavailable for submissions. Nevertheless, the review adhered to a strict, pre-defined internal protocol to ensure methodological rigor.

Literature Search

To ensure a thorough evidence synthesis, systematic searches were conducted in PubMed, Web of Science, Scopus, Cochrane, and Embase up to December 2025. The full, unabridged Boolean search strings utilized for all databases are provided in the Appendix to ensure complete replicability. The strategy incorporated a mix of MeSH terminology and specific keywords ranging from “lumbar segmental instability” to “clinical special tests,” integrated via Boolean operators. This approach allowed for a targeted yet broad exploration of both clinical and radiographic assessment methods. In addition to the electronic database queries, manual hand-searching (citation chaining) of the reference lists of all included full-text articles and previously published reviews was conducted to identify any relevant studies not captured by the initial algorithmic search.

Following initial database screening, full-text articles were retrieved utilizing comprehensive institutional access to paywalled journals, supplemented by open-source repositories. No restrictions were placed on publication access type (i.e., open-access vs. subscription-based). Articles were only excluded during the final eligibility phase if the full text remained completely unobtainable after exhaustive retrieval efforts, ensuring that the qualitative synthesis was based on complete methodological data.

Selection criteria

Inclusion Criteria

We included English-language studies featuring human participants of all ages and genders. The primary inclusion requirement was the application of a diagnostic model for lumbar instability, whether for the purposes of clinical identification, risk prediction, or specialized imaging analysis.

Exclusion Criteria

Exclusion criteria consisted of non-English studies, animal models, and studies investigating artificial intelligence (AI) or machine learning diagnostic algorithms. We also excluded case reports, case series, and conference abstracts lacking full-text availability. To ensure data integrity, duplicate publications and overlapping datasets were removed (detailed eligibility criteria are provided in Table 1).

**Table 1:** Eligibility criteria of the included studies.

Criteria	Inclusion	Exclusion
Study population	Human participants (all genders and ages)	Animal studies
Study design	Full-text original studies	Abstracts lacking full-text access, as well as case reports and case series
Medical condition	Studies targeting patients with lumbar instability.	Research isolated to lumbar canal stenosis and other neurological spine disorders
Technology focus	Studies utilizing clinical, radiographic, or biomechanical evidence of lumbar instability.	Studies without a standard criterion for lumbar instability or studies evaluating AI/machine-learning automated diagnostic models
Data integrity	Primary research and original datasets.	Redundant studies and overlapping data cohorts

Study Selection and Data Extraction

Following PRISMA guidelines and according to the inclusion criteria, two reviewers independently screened all articles. Discrepancies were resolved via consensus or consultation with a third author. Inter-reviewer reliability was quantified using Cohen’s kappa statistic.

Quality Assessment

Methodological quality and risk of bias were appraised using study-specific validated tools to accommodate the heterogeneity of the included study designs. Diagnostic validity studies were evaluated using the Quality Assessment of Diagnostic Accuracy Studies (QUADAS-2) tool [10], while observational cohorts were assessed using the Newcastle-Ottawa Scale (NOS) [11,12]. Finally, the PROBAST (Prediction model Risk of Bias Assessment Tool) [13] was utilized to assess studies involving multivariable prediction models, encompassing both the development of novel scoring systems and the clinical validation of prediction rules in patient cohorts.

Data Synthesis

Due to the substantial clinical and methodological heterogeneity across the included studies, ranging from manual provocative tests to dynamic fluoroscopy, a quantitative meta-analysis was deemed inappropriate. Instead, a thematic narrative synthesis was conducted in accordance with the Synthesis Without Meta-analysis (SWiM) reporting guidelines. Extracted data were systematically categorized and analyzed across three predefined clinical domains: (1) individual clinical examinations and clustered special tests, (2) dynamic imaging and kinematic assessments, and (3) predictive models and clinical scoring systems.

Results

**Figure 1:**
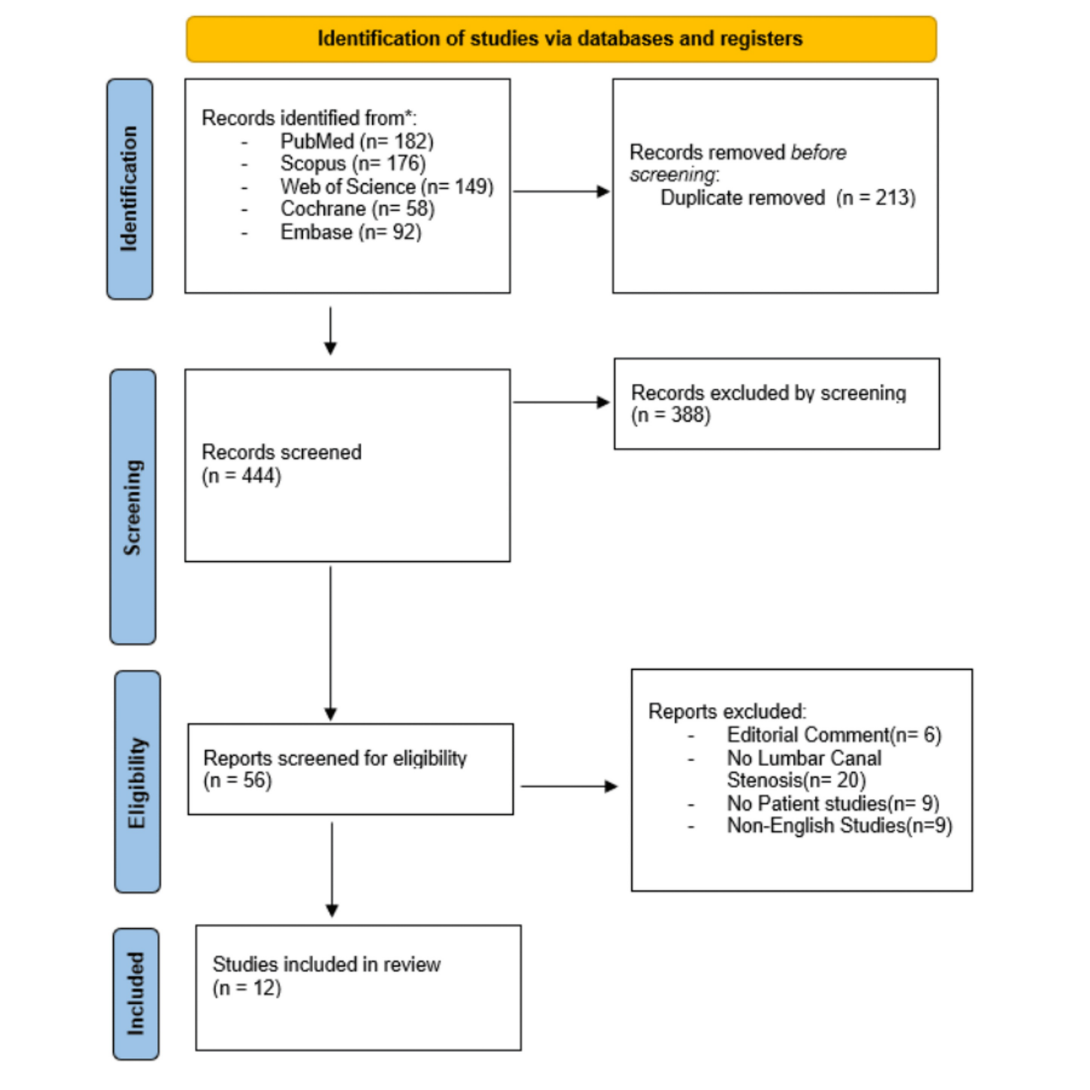
PRISMA flow diagram. PRISMA: Preferred Reporting Items for Systematic Reviews and Meta-Analyses.

Initial database queries yielded 657 records. Following the removal of 213 duplicates, we conducted a two-stage screening of titles, abstracts, and full texts. Initial screening of the 444 records demonstrated substantial inter-reviewer agreement, with a Cohen’s kappa of 0.84. For the full-text eligibility phase (n=56), the agreement was almost perfect (k= 0.91). Ultimately, 12 studies [14-25] satisfied all eligibility criteria for inclusion. The selection process is detailed in the PRISMA flow diagram (Figure 1).

Overall, the included studies demonstrated significant variability in diagnostic approaches. Clinical examination studies frequently highlighted the Passive Lumbar Extension (PLE) test and targeted multi-test clusters (e.g., Apprehension Sign combined with the Prone Instability Test) as highly specific tools for identifying functional instability. Radiographic and observational studies emphasized the utility of dynamic imaging, such as sit-to-stand kinematics and weighted-flexion radiographs, for unmasking mechanical instability that static resting images often miss. Methodological quality across these primary diagnostic studies was generally high, though early predictive models demonstrated a higher risk of statistical bias. The details of the 12 included articles are summarised in Table 2.

**Table 2:** Baseline attributes and key findings of the included studies. CPR: clinical prediction rule; RCT: randomized controlled trial; LRT: lumbar rocking test; PLE: passive lumbar extension test.

Author	Type of Study	Sample Size	Outcome	Population	Results	Analysis
Siribumrungwong et al. (2025) [14]	Diagnostic accuracy	46 were involved in the diagnostic evaluation	Segmental sagittal displacement	Suspected lumbar instability	The 3-kg weight-lifting flexion radiograph significantly increased sensitivity for detecting lumbar instability at L3/4 (88%) and L4/5 (83.3%) levels compared to conventional flexion radiographs	Sensitivity and accuracy of weighted vs. non-weighted imaging
Saleh et al. (2024) [15]	Methodological study (score development)	54 studies were synthesized in the prediction model	Develop Jakarta Instability Score	Patients with degenerative lumbar stenosis	The scoring system effectively identified instability and the need for fusion	Promising clinical tool; requires external validation
Seyedhoseinpoor et al. (2022) [16]	Diagnostic accuracy study	202 participants were involved in the diagnostic cohort	Evaluate combined clinical exams	Subjects experiencing pain in the lower back	A combination of tests improved the prediction of radiographic instability	Supports the use of clinical test clusters for better diagnostic precision
Jiang et al. (2022) [17]	Observational radiographic study	78 individuals were involved in the radiographic analysis	Evaluate dynamic lumbar motion (sit-to-stand test)	Patients with degenerative spondylolisthesis	Dynamic motion differences detected instability effectively	Functional imaging provides better insight than static imaging
Chatprem et al. (2021) [18]	Diagnostic validity study	140 participants were involved in the validity study	Develop a clinical diagnostic tool	Individuals with lumbar instability	The tool showed good validity and reliability	Useful for screening but needs wider validation
Areeudomwong et al. (2020) [19]	Cross-sectional diagnostic study	200 participants were involved in the validity study	Diagnostic utility of clinical test clusters	Patients with chronic low back pain (CLBP)	Apprehension Sign (AS) has high specificity (92.6%); a cluster of 3 tests provides a powerful support tool	Sensitivity, specificity, and likelihood ratio (LR+) analysis
Rathod et al (2019) [20]	Diagnostic clinical study	50 participants were involved in the clinical assessment	To assess the effectiveness of the Lumbar Rocking Test (LRT) in predicting lumbar instability	Subjects experiencing chronic pain in the lower back	LRT showed good sensitivity and specificity in identifying lumbar instability (95.56% and 40%)	LRT is a simple, noninvasive clinical test with good diagnostic value; however, it should be used alongside other clinical and radiological assessments for accurate diagnosis
Yeo et al. (2018) [21]	Observational study	75 individuals were involved in the clinical analysis	Evaluate the necessity of dynamic radiographs in diagnosing lumbar segmental instability)	Patients with suspected lumbar segmental instability	Dynamic radiographs were not always required; clinical assessment and static imaging were often sufficient	Suggests limited added value of dynamic radiographs; supports reliance on clinical judgment and selective imaging
Esmailiejah et al. (2018) [22]	Cross-sectional diagnostic study	52 individuals were involved in the diagnostic evaluation	Assess the diagnostic accuracy of clinical tests	Patients with suspected lumbar instability	Individual tests had moderate accuracy; combined tests improved diagnosis	No single test is sufficient; a combination increases diagnostic validity
Rabin et al. (2013) [23]	Randomized Controlled Trial	105 participants	Validation of a clinical prediction rule (CPR) for stabilization	Patients experiencing nonspecific low back pain	The original 4-item CPR was not validated; however, a modified 2-item CPR (positive prone instability test and aberrant movement) successfully identified patients highly responsive to lumbar stabilization	RCT interaction analysis assessing treatment response against CPR status
Ahmadi et al. (2009) [24]	Experimental kinematic study	30 participants were involved in the kinematic study	Analyze lumbar motion using video fluoroscopy	Patients with lumbar instability	Abnormal motion patterns identified in unstable segments	Dynamic assessment is crucial for accurate diagnosis
Kasai et al. (2006) [25]	Diagnostic clinical study	122 participants were involved in the validation study	Evaluate the passive lumbar extension (PLE) test	Patients with low back pain	The PLE test demonstrated high diagnostic accuracy (sensitivity: 84.2%, specificity: 90.4%)	PLE is a simple, reliable screening tool, but it requires confirmation with imaging

Quality Assessment

Diagnostic accuracy studies (QUADAS-2): Risk of bias for the diagnostic studies was low to moderate. However, the reported diagnostic accuracy metrics must be interpreted as indicative findings rather than absolute values, as the included studies (ranging from n = 46 to n = 202) did not report 95% confidence intervals. The absence of these intervals makes it difficult to gauge the precision of the reported sensitivity and specificity estimates. Unclear reporting regarding reference standards was the primary limitation for Kasai et al., Esmailiejah et al., and Chatprem et al., whereas Seyedhoseinpoor et al., Rathod et al., Areeudomwong et al., and Siribumrungwong et al. demonstrated the most robust methodology across all domains. Applicability concerns were consistently low across all seven studies for patient selection, index testing, and reference standards, indicating exceptionally high clinical relevance for the targeted diagnostic maneuvers (Table 3).

**Table 3:** Quality Assessment for Diagnostic Accuracy Studies (QUADAS-2).

Author	Patient Selection	Index Test	Reference Standard	Flow and Timing	Overall Risk
Siribumrungwong et al. (2025) [14]	Low	Low	Low	Low	Low risk
Seyedhoseinpoor et al. (2022) [16]	Low	Low	Low	Low	Low risk
Areeudomwong et al. (2020) [19]	Low	Low	Low	Low	Low risk
Rathod et al. (2019) [20]	Low	Low	Low	Low	Low risk
Kasai et al. (2006) [25]	Low	Low	Unclear	Low	Low risk
Chatprem et al. (2021) [18]	Low	Unclear	Unclear	Low	Moderate risk
Esmailiejah et al. (2018) [22]	Low	Unclear	Unclear	Low	Moderate risk

Observational studies (Newcastle-Ottawa Scale (NOS)): Among the observational studies, methodological quality ranged from Fair to Good. It is important to note that these cohorts were relatively small, such as Ahmadi et al. (n=30) and Yeo et al. (n=75), and the lack of reported confidence intervals suggests that these findings represent clinical trends in functional instability that require further high-powered validation. Jiang et al. [17] demonstrated superior quality, recording the highest score, reflecting a robust methodology in evaluating dynamic lumbar motion using the sit-to-stand test. Ahmadi et al. [24] and Yeo et al. [21] each scored a fair rating, indicating moderate quality primarily due to minor limitations in comparability controls or isolated outcome assessments (Table 4).

**Table 4:** Quality assessment for observational studies (Newcastle-Ottawa Scale). Quality ratings are based on standard thresholds established by the Agency for Healthcare Research and Quality (AHRQ) [12]: Good: 3–4 stars in Selection and 1–2 stars in Comparability and 2–3 stars in Outcome. Fair: 2 stars in Selection and 1–2 stars in Comparability and 2–3 stars in Outcome. Poor: 0–1 star in Selection or 0 stars in Comparability or 0–1 star in Outcome.

Author	Selection (max 4)	Comparability (max 2)	Outcome (max 3)	Total Score( 0- 9)	Overall Rating
Jiang et al. (2022) [18]	4	1	2	7	Good
Yeo et al. (2018) [21]	3	1	2	6	Fair
Ahmadi et al. (2009) [25]	3	1	2	6	Fair

Prediction models (PROBAST): Both included clinical prediction models (Saleh et al. [15] and Rabin et al. [23]) exhibited an overall high risk of bias according to strict PROBAST criteria. While these models offer promising diagnostic frameworks, their high-risk status, compounded by the lack of reported confidence intervals and underpowered sample sizes, necessitates that their findings be framed as preliminary evidence. However, it is critical to note that both studies demonstrated robust methodology with low concerns regarding patient selection, predictors, and outcome assessments; they were downgraded exclusively within the statistical analysis domain. This is an expected methodological limitation, as Saleh et al. [15] developed a novel scoring system (the Jakarta Instability Score) that currently lacks independent external validation, while Rabin et al. [23] utilized a standard physical therapy randomized controlled trial (RCT) cohort (n=105) that falls below the massive epidemiological event-per-variable statistical thresholds strictly required by PROBAST modeling (Table 5).

**Table 5:** Risk of bias for prediction model studies with Prediction Model Risk of Bias Assessment Tool (PROBAST).

Author	Participant Selection	Predictors	Outcome	Analysis	Overall Risk of Bias
Saleh et al. (2024) [15]	Low	Low	Low	High	High risk
Rabin et al. (2013) [23]	Low	Low	Low	High	High risk

Discussion

This review analyzes 12 studies and critiques current lumbar instability detection methods while identifying emerging diagnostic strategies. Lumbar segmental instability is a complex clinical disorder characterized by abnormal vertebral segment motion that can lead to pain, impaired function, and potential neurological involvement. Accurately diagnosing this condition is critical, as it directly influences fundamental treatment decisions, guiding clinicians in choosing between conservative management strategies, such as targeted stabilization exercises and bracing, and surgical interventions like spinal fusion.

Summary of Main Findings: Clinical Diagnosis

Contemporary literature emphasizes the distinction between mechanical (radiographic) instability and functional (clinical) instability. Diagnosing functional instability, which involves aberrant movement patterns and motor control deficits, remains a significant clinical challenge. Symptoms include mechanical pain over the lower back sensitive to extension and static loading, frequently accompanied by subjective reports of lumbar instability, "giving way" or "catching" during spinal transitions.

In terms of clinical examination, Kasai et al. reported that the Passive Lumbar Extension (PLE) test is a highly reliable screening tool, with a sensitivity of 84.2% and a specificity of 90.4% for detecting instability, though it requires subsequent confirmation via imaging [25]. In addition to current evidence-based options, our review evaluated the Lumbar Rocking Test (LRT) introduced by Rathod et al. [20]. His high-quality evidence underscores the LRT as a highly valid clinical maneuver, yielding a sensitivity of 95.56% and a specificity of 40%, serving as an alternative or complement to the PLE test, particularly for patients who may struggle with extension-based assessments [20,25].

However, relying on a single clinical test is often inadequate. Esmailiejah et al. demonstrated that individual clinical tests possess only moderate diagnostic accuracy on their own when compared to an intraoperative "gold standard" [22]. To overcome this limitation, clustered testing is highly recommended. Supporting this, Areeudomwong et al. demonstrated that while the Apprehension Sign alone possesses high specificity (92.6%), utilizing a targeted cluster of three clinical tests (Apprehension Sign, Instability Catch Sign, and Prone Instability Test) provides a powerful diagnostic support tool with a high positive likelihood ratio (LR+ 5.80) for ruling in functional instability [19]. Furthermore, a recent 2025 scoping review by Tsartsapakis et al. synthesized the reliability and validity of manual tests for functional lumbar segmental instability [26]. Their findings reinforce that while individual maneuvers are useful, comprehensive subclassification and clustered manual testing provide a far more robust clinical picture. This aligns with foundational diagnostic accuracy work by Fritz et al., who established that the presence of at least 53 degrees of lumbar flexion, or a lack of hypomobility with intervertebral motion testing, yielded a positive likelihood ratio of 4.3 (95% CI: 1.8 to 10.6) for accurately predicting radiographic instability based on clinical examination alone [8]. 

Seyedhoseinpoor et al. demonstrated that combining multiple clinical exams significantly improves the prediction of radiographic instability, thereby offering greater diagnostic precision [16]. Similarly, Chatprem et al. successfully developed a valid and reliable clinical diagnostic tool tailored for screening individuals with lumbar instability, although it requires broader external validation [18].

Comparison With Existing Literature: Imaging Modalities

Mechanical instability is objectively measured via imaging, typically quantified through translational and angular displacement, but controversies persist regarding the most effective radiological approach.

To capture true functional deficits, dynamic assessments are increasingly favored over static resting images. Ahmadi et al. used digital video fluoroscopy for kinematic analysis, successfully identifying abnormal motion patterns in unstable segments and demonstrating that dynamic assessment is crucial for accurate diagnosis [24]. Jiang et al. further supported this by showing that functional imaging of dynamic lumbar motion, specifically during a five-repetition sit-to-stand test, detected instability more effectively than static imaging [17]. Notably, Esmailiejah et al. also reported that dynamic radiography yielded an accuracy rate exceeding 90% when validated against intraoperative findings [22].

To further refine these imaging techniques, recent advancements highlight the development of fully automated radiographic metrics. Hipp et al. [27] demonstrated that these emerging technologies aim to quantify sagittal plane translational and vertical instabilities from dynamic X-rays without human error, significantly reducing the inter-observer variability that currently plagues manual radiological measurements. Furthermore, to enhance the sensitivity of functional radiographs, Siribumrungwong et al. [14] demonstrated that utilizing a 3-kg weight-lifting flexion radiograph significantly increases the detection rate of sagittal lumbar instability at the L3/L4 (88%) and L4/L5 (83.3%) levels compared to conventional, unweighted dynamic imaging [15]. Conversely, Yeo et al. cautioned against the overutilization of specialized imaging, arguing that dynamic radiographs are not universally essential; they found that sound clinical assessment combined with standard static imaging is often sufficient for diagnosis [21].

Implications for Practice and Future Research: Predictive Models.

Accurate diagnosis of lumbar segmental instability is only valuable if it directs appropriate intervention. Rabin et al. [23] demonstrated this by validating a clinical prediction rule (CPR) for stabilization exercises. Their randomized controlled trial revealed that while an original four-item CPR was not validated, a modified two-item CPR, requiring only a positive prone instability test and the presence of aberrant movements, successfully identified patients who were highly responsive to targeted lumbar stabilization exercises. However, it is critical to note that Rabin et al. utilized a standard physical therapy randomized controlled trial (RCT) cohort (n=105), which falls below the massive epidemiological event-per-variable statistical thresholds strictly required by PROBAST modeling. Consequently, while this streamlined rule provides clinicians with a practical, evidence-based tool for triaging non-operative care, its high risk of statistical bias necessitates that these findings be framed as preliminary evidence. Saleh AK et al. introduced the Jakarta Instability Score [15]. While conceptually interesting, this tool received a High Risk of bias rating according to PROBAST criteria. Because it lacks independent external validation, the Jakarta Instability Score must be viewed strictly as an experimental framework. It currently possesses no clinical utility for guiding invasive procedures such as spinal fusion.

Beyond this specific score, the broader literature reflects a growing shift toward advanced predictive modeling to bridge the gap between imaging and surgical planning. For instance, Kao et al. developed a preoperative predictive model utilizing three-dimensional computed tomography to anticipate lower lumbar spine instability, reporting a high diagnostic performance with a sensitivity of 88.7%, specificity of 93.1%, and an overall predictive accuracy of 88.2% [28]. Similarly, Zhang et al. utilized LASSO regression to build a diagnostic model specifically for radiographic instability in L4-5 degenerative spondylolisthesis [29]. Their multifactor model yielded an outstanding Area Under the Curve (AUC) of 0.972, with a sensitivity of 86.46% and a specificity of 95.19%. While these models represent the future of personalized surgical planning, they share the same limitations as the Jakarta score: they remain experimental frameworks that require robust, multicenter external validation before they can be safely integrated into definitive clinical guidelines.

Strengths and Limitations of the Review

A persistent barrier in the field is the theoretical understanding of instability itself. Panjabi's neutral-zone model remains the foundational biomechanical framework for this condition, correctly conceptualizing instability as increased laxity arising from dysfunction in the active, passive, and neural subsystems. However, contemporary biomechanical literature has refined this 1990s paradigm. Recent in vivo kinematic studies highlight that translating this theoretical laxity into objective, measurable clinical criteria remains difficult, necessitating advanced motion-capture and dynamic imaging to distinguish true mid-range motor control deficits from purely structural tissue failure.

Finally, while the inclusion of 12 primary studies may appear modest relative to the initial search yield, this stringent filtering underscores a critical finding of the review itself: there is a profound paucity of high-quality, primary diagnostic studies utilizing standardized criteria for lumbar segmental instability. The high exclusion rate was driven by rigorous adherence to PRISMA guidelines and by deliberately prioritizing internal validity over volume. While many included studies appropriately evaluated broad patient populations (such as those with chronic non-specific low back pain) to establish baseline diagnostic metrics, we strictly excluded studies that failed to apply a robust reference standard to objectively differentiate true clinical instability from general mechanical pain within those cohorts. Consequently, this review provides a highly distilled, rather than simply exhaustive, synthesis of the most methodologically sound evidence currently available.

Furthermore, the statistical power of several key findings in this review is constrained by small cohort sizes. Foundational studies, such as those by Ahmadi et al. (n=30) [24], Siribumrungwong et al. (n=46) [14], and Rathod et al. (n=50) [20], utilize samples that may be underpowered for broad diagnostic-accuracy research. This limitation is compounded by a general absence of reported confidence intervals (CIs) across the included literature. Without CIs, the precision and reliability of the reported sensitivity and specificity estimates remain difficult to gauge, as small variations in participant outcomes could significantly shift the reported diagnostic values. Consequently, the accuracy figures presented in this review should be viewed as indicative rather than absolute.

## Conclusions

The diagnosis of lumbar segmental instability remains a multifaceted challenge that cannot be adequately addressed by a single clinical test or imaging modality. This systematic review highlights that while the PLE test and dynamic functional imaging (such as video fluoroscopy and sit-to-stand kinematics) represent the most accurate individual tools currently available, they are most effective when utilized as part of a comprehensive, multimodal diagnostic strategy. While clinical test clustering provides some utility for conservative management, emerging predictive models intended for surgical decision-making, such as the Jakarta Instability Score, demonstrate a high risk of bias and a critical lack of external validation. Consequently, these scoring systems cannot be recommended for standardizing patient assessment, and they should under no circumstances be used to guide definitive surgical interventions, such as spinal fusion, at this time.

However, the field is still hindered by a lack of universally accepted diagnostic criteria and the conceptual overlap between mechanical and functional instability. To bridge the gap between current diagnostic limitations and objective treatment decisions, future research must prioritize prospective, multicenter studies and pre-registered protocols. Furthermore, establishing a universally standardized imaging reference criterion is essential before integrated scoring systems or automated metrics can be safely translated into routine clinical practice.

## Appendices

Search strategies

PubMed/MEDLINE

("lumbar segmental instability"[Title/Abstract] OR "lumbar segmental instability"[Title/Abstract] OR "lumbar instability"[Title/Abstract]) AND ("clinical diagnosis"[Title/Abstract] OR "diagnostic tests"[Title/Abstract] OR "diagnosis"[MeSH Terms] OR "clinical examination"[Title/Abstract] OR "special tests"[Title/Abstract] OR "imaging"[Title/Abstract] OR "radiography"[Title/Abstract] OR "radiography"[MeSH Terms] OR "MRI"[Title/Abstract] OR "magnetic resonance imaging"[MeSH Terms]) AND (English[Language]) NOT ("animal"[MeSH Terms] NOT "human"[MeSH Terms]) NOT ("artificial intelligence"[Title/Abstract]).

Web of Science

TS=("lumbar segmental instability" OR "lumbar segmental instability" OR "lumbar instability") AND TS=("clinical diagnosis" OR "diagnostic tests" OR "clinical examination" OR "special tests" OR "imaging" OR "radiography" OR "MRI" OR "magnetic resonance imaging") NOT TS=("artificial intelligence" OR "machine learning" OR "AI") AND LA=(English).

Scopus

TITLE-ABS-KEY("lumbar segmental instability" OR "lumbar segmental instability" OR "lumbar instability") AND TITLE-ABS-KEY("clinical diagnosis" OR "diagnostic tests" OR "clinical examination" OR "special tests" OR "imaging" OR "radiography" OR "MRI" OR "magnetic resonance imaging") AND NOT TITLE-ABS-KEY("artificial intelligence" OR "deep learning" OR "machine learning") AND LIMIT-TO(LANGUAGE, "English") AND LIMIT-TO(EXACTKEYWORD, "Human").
